# Can Green Technological Innovation Reduce Hazardous Air Pollutants?—An Empirical Test Based on 283 Cities in China

**DOI:** 10.3390/ijerph19031611

**Published:** 2022-01-30

**Authors:** Ning Ma, Puyu Liu, Yadong Xiao, Hengyun Tang, Jianqing Zhang

**Affiliations:** 1 Institute of Central China Development, Wuhan University, Wuhan 430072, China; madison@whu.edu.cn (N.M.); pclpy001@whu.edu.cn (P.L.); 2 Institute of Regional and Urban-Rural Development, Wuhan University, Wuhan 430072, China; 3 School of Economics and Management, Wuhan University, Wuhan 430072, China; ydxiao@whu.edu.cn

**Keywords:** green science, technological innovation, hazardous air pollutants, spatial spillover effect, mediating effect

## Abstract

Based on the panel data of 283 cities in China from 2009 to 2018, this paper analyzes the effect of urban green scientific and technological innovation enhancement on hazardous air pollutants using the GS2SLS method, which simultaneously controls for model endogeneity and spatial spillover effects and reveals the transmission mechanism of urban green scientific and technological innovation level. It was found that (1) There is a significant spatial spillover effect of hazardous air pollutants between regions, both in China as a whole and in the eastern, central, and western parts of the country, and the spatial spillover effect of hazardous air pollutants is significantly greater in the eastern and central parts of China than in the western parts. (2) Green technological innovation has a significant inhibitory effect on hazardous air pollutants in cities in eastern and central China. An extended study found that the improvement in green technology levels in innovative cities has a better effect on controlling hazardous air pollutants than in non-innovative cities. (3) The energy- saving and green economy effects have a mediating influence on the effect of green technological innovation on hazardous air pollutants in cities, and the simultaneous occurrence of these two effects in green technological innovation serves to enhance the transmission of hazardous air pollutants in order to facilitate the long-term management of haze.

## 1. Introduction

### 1.1. Background

Since the industrial revolution, the world has been experiencing rapid economic growth accompanied by global warming, rising sea levels, and increasing environmental pollution. These problems are serious threats to human health and to economic and social development [1,2,3]. In China, along with rapid urbanization, the massive burning of fossil energy, rapid economic growth, and hazardous air pollutants, of which PM is an integral component, have frequently been occurring, and the scope of pollution has been expanding and deepening. This has been seriously threatening the daily lives and health of most urban residents [4,5,6]. In recent years, the Chinese government has implemented a series of measures to combat hazardous air pollutants to win the “Blue Sky Defense War” and has achieved certain results in the treatment of air pollution. However, overall air quality still needs to be improved [7,8,9]. According to China’s Ecological Environment Status Bulletin (2018), only 157 out of 337 cities in China meet the ambient air quality standards, while 180 cities fall below these standards (53.4% of all cities); the number of days with PM_2.5_, which is the main pollutant, accounts for 45% of heavily polluted days and above [10]. Therefore, effectively combating air pollution while ensuring sustainable economic development is a concern shared by all sectors of Chinese society.

Scientific and technological innovations are necessary to improve the efficiency of resource usage and reduce pollutant emissions, and they are of great significance in solving environmental pollution problems [11]. However, an increase in the level of technological innovation does not always lead to a reduction in environmental pollution [12]. In reality, as China’s environmental protection policies become stricter, many industrial enterprises are gradually increasing their independent research and development expenditures. They aim to change the excessive reliance on energy consumption, which is objectively beneficial for environmental improvement. However, for traditional industrial sectors using fossil fuels, increasing investment in scientific and technological innovation will undoubtedly expand their scale while also causing greater environmental pollution [13,14]. Therefore, green technological innovation-oriented clean technologies have emerged [15]. Green technology refers to a general term for technologies that follow ecological principles and ecological and economic laws, conserve resources and energy, establish an environment conducive to the protection of human survival and health, reduce the costs of production and consumption behavior, and create sustainable development as their core function [16]. Green scientific and technological innovation includes the development and utilization of new energy, waste utilization technology, environmental engineering technology, and a series of science and technologies [17,18,19,20]. Green scientific and technological innovation tries to achieve the effect of saving energy and reducing emissions through technological upgrading to change the high pollution and high energy consumption of the industrial structure’s system and innovate a front-end production system for enterprises [21,22].

**Figure 1:**
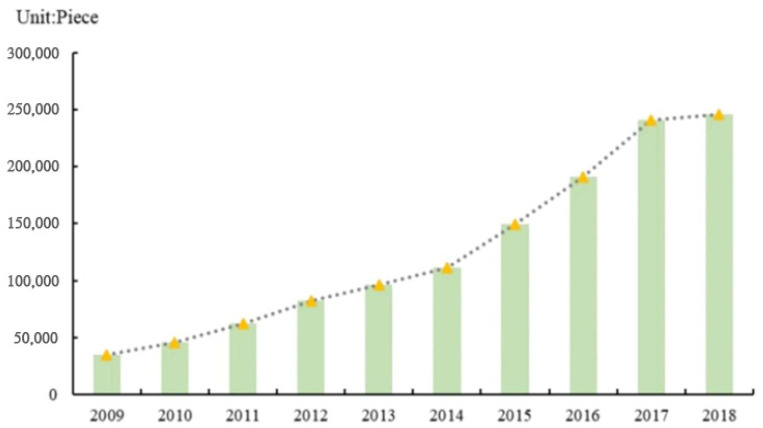
Total number of green patent applications in Chinese cities, 2009–2018.

At present, China is very active in green scientific and technological innovation activities, and the innovation capacity of green technology is constantly improving [23,24]. Figure 1 shows the total number of green patent applications in Chinese cities from 2009–2018. The number has steadily increased. However, pressure on ecological and environmental protection remains high because of the influence of the economic and energy structure as well as the continued uncertainty caused by COVID-19 on economic and social development. Therefore, will the growing number of green invention patents in China help combat hazardous air pollutants? What are the transmission pathways that affect hazardous air pollutants? Given China’s vast territory, each region has its own innovation resource endowment and economic development level. Is the impact of green technological innovation on smog pollution heterogeneous in different regions of China?

In view of this background, this study used the panel data of 283 Chinese cities from 2009 to 2018 and measured the level of green scientific and technological innovation using the total number of green patent applications. We used PM_2.5_ to refer to the degree of hazardous air pollutants and analyzed the impact of green scientific and technological innovation on hazardous air pollutants through generalized spatial two-stage least squares (GS2SLS). The GS2SLS method can simultaneously control for spatial spillover effects and model the endogeneity mechanism. The marginal contributions of this study are mainly reflected in the following three aspects: (1) Compared with previous studies, this study identified the number of green patent applications from the number of innovation patent applications and selected the total number of green patent applications to accurately measure the level of green scientific and technological innovation and the green innovation capability of cities. (2) Based on a testing principle of the mediation effect, this paper constructed the mediation effect model composed of three regression equations to accurately identify the transmission mechanism of the urban green innovation technology level in promoting the control of hazardous air pollutants. We hoped to provide a reasonable and effective experience for controlling national and regional hazardous air pollutants. (3) A systematic study of the impact mechanisms of hazardous air pollutants was conducted from a new perspective on urban green technology upgrading. Compared with previous studies, 283 cities in China were considered as the research objects. The heterogeneity of green technological innovation regarding hazardous air pollutants in innovative and non-innovative cities is further discussed.

### 1.2. Literature Review and Theoretical Analysis

#### 1.2.1. Innovation and Pollution

The technical advancement or innovation in pollution control is, and will remain, a very important factor affecting the success of efforts that are made to improve environmental quality and maintain growth of output. Omri and Hadj (2020) found that FDI inflows have positive effects on the four indicators of carbon emissions, while increasing governance quality and technological innovation have negative effects on these indicators [25]. Carrion-Flores and Innes (2010) showed that tightened pollution targets induce environmental innovation, but the ‘‘environmental policy multiplier’’—the proportionate contribution of induced innovation to long-run emissions reduction—is small [26]. In Malaysia, Sohag et al. (2015) showed that technological innovation significantly reduces energy consumption, which in turn reduces CO_2_ emissions [27]. Using the data from 25 Sub-Saharan African countries over the 1996–2010 period, Abid (2016) examined the factors affecting CO_2_ emissions. He confirmed the importance of governance indicators in lowering CO_2_ emissions [28]. Zhang et al. (2016) applied a Malmquist technique to analyze the effect of technological innovation on CO_2_ emissions in the case of 38 industrial sectors. They found that technological innovation boosted the performance of carbon emissions over the studied period [29].

#### 1.2.2. Impact Factors of Hazardous Air Pollutants

Over the last two decades, regional air pollution has attracted extensive attention from researchers, both domestically and internationally. A review of the existing literature reveals that current research on the impact mechanism of hazardous air pollutants mainly focuses on three aspects: economic, policy, and innovation factors. (1) Economic factors: Existing studies have found that industrial agglomeration and international trade brought about by economic development are some of the main reasons for the increase in hazardous air pollutants. Among them, Frank (2001) found a significant positive correlation between the scale of industrial agglomeration and air pollution in a sample of 200 urban agglomerations in the EU [30]. Virkanen (1998) showed that industrial agglomeration in southern Finland is the direct cause of a large amount of air and water pollution [31]. Dietlzenbacher et al. (2012) found that each additional dollar of China’s export trade would emit 34% more carbon dioxide than the normal export trade [32]. (2) Policy factors: Existing studies have found that the government’s environmental regulation policies would have a significant impact on hazardous air pollutants. For example, Zheng et al. (2013) found that under the central and public requirements and expectations of local governments to strengthen environmental protection, energy conservation and emissions reductions have become the assessment basis affecting the promotion of local officials, in addition to economic growth [33]. (3) Innovation factors: Most researchers have focused on the impact of technological innovation on hazardous air pollutants, but this impact is two-sided. On the one hand, technological innovation helps mitigate environmental pollution; for example, Grossman et al. (1995) found that technological progress reduces environmental pollution [34]. Liu (2018) found that technological innovation not only reduces hazardous air pollutants but also indirectly reduces the level of haze in neighboring provinces through knowledge spillover effects [35]. On the other hand, technological innovation can also aggravate environmental pollution. Wang (2020) used spatial econometric analysis tools to prove that an increase in investment in scientific and technological innovation does not necessarily lead to the improvement in environmental quality and that R&D investment is negatively correlated with environmental quality [36].

### 1.3. Hypothesis

From the perspective of green technological innovation, this paper studied the mechanism of its effect on hazardous air pollutants and on haze reduction mainly through the following two ways to analyze the direct inhibiting effect on hazardous air pollutants. (1) Promoting sustainable economic development. Green technological innovation is no longer limited to simply reducing production costs and improving economic efficiency. It also emphasizes the establishment of a management model and control mechanism that coordinate the economy, the resources, and the environment, “forcing” producers to include resource and environmental costs in their production costs. This promotes in-depth research and development in related fields and in the effective supply of ecological and environmental protection products. Moreover, it continues to trigger the green transformation of various production organizations in terms of development strategies, products and services, and organizational systems, thereby promoting the construction of a green, efficient, and low-carbon production system [37]. This will transform the crude development model of high input and high consumption, achieve the coordinated development of the economy, the resources, and the environment, promote sustainable economic development, and thus reduce hazardous air pollutants. (2) Improving green competitiveness of enterprises with the continuous improvement in the environmental regulations and environmental standards of production systems; an increase in environmental requirements can inspire enterprises to expand their green business and gain competitive advantages in the market by seeking green technological innovations. To gain more market opportunities and expand market share, an increasing number of enterprises will expand their green scientific and technological innovation chain by increasing capital, intellectual, and equipment investment and increasing the supply of quality products that meet ecological and environmental standards [38]. Therefore, green scientific and technological innovation can force enterprises to engage in clean production and mitigate hazardous air pollutants by enhancing their competitiveness. Based on the above analysis, the first hypothesis of this study was proposed.

**Hypothesis** **1.** *Increased levels of green technology and innovation have an abatement effect on hazardous air pollutants.*

Green technological innovation may also have an indirect effect on hazardous air pollutants through mediating factors. (1) The green technology progress effect. Green technological innovation capacity helps accelerate the growth of green technology in cities; research shows that green technological innovation causes the promotion of regional green total factor productivity and is also an endogenous mechanism to promote sustainable economic growth transformation [39]. Green technological innovation is conducive to improving energy efficiency and reducing carbon emissions. Green total factor productivity is considered an environmental performance metric; green total factor productivity growth is largely dependent on the development of green innovation. Therefore, green scientific and technological innovation can promote regional green technological progress and influence pollution emissions in the production process of enterprises. (2) Energy-saving effects. China’s green technological innovation activities are mainly active in five technology fields: pollution management, environmental materials, alternative energy, energy-saving, and emissions reduction. The application of green technological innovation in the energy field helps enterprises to adopt more clean energy and renewable energy in the production process and reduce the use of fossil fuels. Most of the hazardous air pollutants comprise gaseous pollutants that originate from the burning of fossil fuels; therefore, technological innovation focuses on reducing the use of fossil fuels and other energy, thus contributing to the abatement of hazardous air pollutants [40]. (3) Industrial structure effects. At present, China’s industrial sector accounts for a high proportion of GNP, and the traditional industrial sector, with high pollution, high emissions, and high consumption, is one of the major reasons for the increase in hazardous air pollutants. Improvement in the level of green technology can promote the progress of production technology, improve the utilization rate of resources and energy in industrial production so that the use of resources can be effectively saved and utilized, and cause pollutant emissions to drop to achieve effective improvement in the environment. The improvement in the urban green technology level expands the potential boundary of production by improving the technical efficiency and scale efficiency of production. This gradually eliminates the pollution-intensive secondary industry [41] and actively develops tertiary industries represented by modern service industries and technology-intensive industries, promotes the transformation of the industrial structure from secondary industries to tertiary industries, achieves the effect of reducing environmental pollution from the overall industrial structure, and reduces the generation of hazardous air pollutants [42]. (4) Green economic effects. Gross domestic product (GDP) is a combination of the market value of all products or services produced by a region or country in a certain period, and it is an important indicator of the level of economic development of a region. According to the Kuznets curve, when a region’s economic development rises rapidly, environmental pollution also rises sharply. Therefore, the blind promotion of the “GDP-only” theory aggravates hazardous air pollutants [43]. Reflecting on China’s crude economic growth model and traditional GDP accounting, the concept of green GDP was proposed. The implementation of green GDP is an inevitable choice for changing China’s economic growth model, which is not only conducive for a better assessment of the performance of local government officials but also for the future development of China’s low-carbon economy [44]. Therefore, improving the level of green technology in cities can reduce environmental pollution from haze by increasing green GDP. In summary, a second hypothesis was proposed.

**Hypothesis** **2.** *The level of urban green innovation influences hazardous air pollutants through the transmission of mediating factors, including the improvement in green science and technology.*

## 2. Materials and Methods

### 2.1. Construction of the Econometric Model

The IPAT model proposed by Ehrlich and Holdren (1971) is widely used as a model for analyzing the impact of human activities on the environment [45]. The basic equation of the model is I = PAT, where I is pollution, P represents the population, A represents the level of affluence, and T is the level of technology. The IPAT model does not allow for non-monotonic, differentially scaled changes in various influencing factors; therefore, its application is greatly limited. To overcome this shortcoming, Dietz and Rosa (1994) developed the IPAT equation into the STIRPAT model [46], which is constructed as shown in Equation (1).

(1) $$I_{i}=a\cdot P_{i}^{b}\cdot A_{i}^{c}\cdot T_{i}^{d}e_{i}$$

Taking the natural logarithm for both sides of the equation:

(2) $$\ln I_{i}=\ln a+b(\ln P_{i})+c(\ln A_{i})+d(\ln T_{i})+\varepsilon_{i}$$

where the subscript i is the unit of observation, a is the constant term, b, c, and d are the parameters to be estimated for each variable coefficient, *e* is the error term, and *ε* is the logarithmic form of *e*. Based on the STIRPAT model, this study first constructed the following benchmark model for hypothesis 1 to examine the impact of innovation efficiency on hazardous air pollutants.

(3) $$\ln PM_{it}=\alpha_{0}+\alpha_{1}\ln E_{it}+\alpha_{2}X_{it}+\varepsilon_{it}$$

where i is a cross-sectional unit of 283 prefecture-level cities in China (as of 2009, there were 298 prefecture-level cities in China, 15 cities located in the western region with missing statistics are excluded here), t denotes the year, PM is the explanatory variable of hazardous air pollutants, E is the core explanatory variable of green technological innovation, X is a set of control and mediating variables, α0–α2 are parameters to be estimated, and ε is a random disturbance term.

### 2.2. Selection of Indicators and Data Sources

#### 2.2.1. Selection of Indicators

(1) Explained variable: hazardous air pollutants (PM_2.5_)

In this study, the annual average PM_2.5_ concentration was used to represent the hazardous air pollutants. The annual average global PM_2.5_ concentration was obtained from the International Earth Science Information Network Centre of Columbia University, and the annual average PM_2.5_ concentration data of each prefecture-level city in China were calculated using ArcGIS software. Haze is mainly composed of fog and haze, the main component of which is water, so it is not polluting in itself. It is caused by the exceedance of various suspended particulate matters in the air, which are mainly composed of sulfur dioxide, nitrogen oxides, and respirable particulate matter. Hazardous air pollutants are part of atmospheric pollution; they are thus a profile of the production and spread of various suspended floating substances in the atmosphere. Hazardous air pollutants mainly consist of sulphur dioxide, nitrogen oxides, and inhalable particles. The first two are mainly gaseous pollutants, and the last item, respirable particulate matter, is the main cause of hazardous air pollutants. Inhalable particles mainly refer to PM_2.5_, which refers to pollutant particles with an aerodynamic equivalent diameter of less than or equal to 2.5 microns [47]. PM_2.5_, as used in this study, refers to fine particulate matter in ambient air with an aerodynamic equivalent diameter of less than or equal to 2.5 microns. It can exist in the air for a longer period, and its higher content in the air concentration represents more serious air pollution.

**Figure 2:**
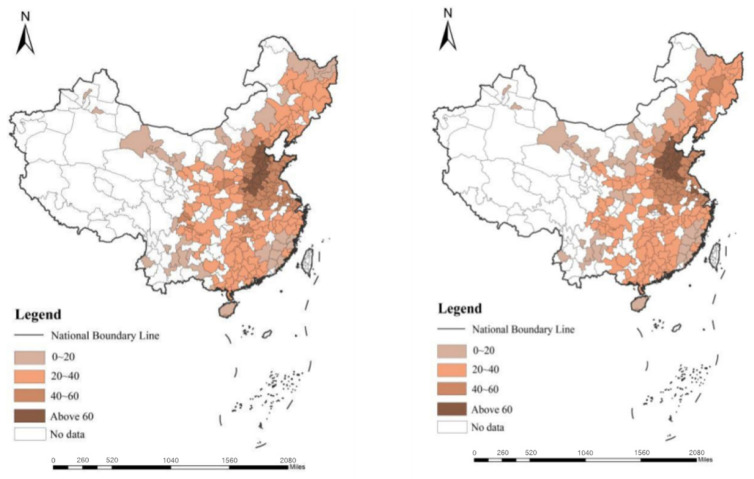
Distribution of hazardous air pollutants in Chinese cities in 2009 and 2018.

ArcGIS software was used to visualize and analyze hazardous air pollutants in 283 Chinese cities. Figure 2 shows the distribution of hazardous air pollutants in Chinese cities from 2009 to 2018, with darker colors representing higher levels of pollution. It can be seen that the distribution of hazardous air pollutants in Chinese cities shows the characteristics of a contiguous and blocky distribution, showing a decreasing pattern from the eastern to the western regions; the areas with serious hazardous air pollutants are mainly concentrated in the North China Plain and the northern coastal areas, and areas with lower pollution levels are mainly distributed in the southwest and the southern coastal areas. Further confirming that hazardous air pollutants are spatially correlated, we could continue to use spatial econometric models to explore the mechanisms by which green technological innovation affects hazardous air pollutants.

(2) Explanatory variable: green technological innovation (E)

Patent data are a valid measure of the level of scientific and technological innovation in a particular field [48,49,50,51,52]. Therefore, the core explanatory variable in this study was green patents, with their definition obtained from the International Patent Green Classification List issued by the World Intellectual Property Organization. Based on this standard, the data on the total number of green patent applications were analyzed using the patent search of the State Intellectual Property Office, with the few missing data being completed by linear interpolation.

(3) Intermediate variables
  1. Green technological progress. This study used green total factor productivity (GFTP) to represent the green technological progress of cities. The GFTP is mainly divided into input and output indicators, where input factors mainly include labor, capital, and energy inputs. Labor input was measured by the number of people employed at the end of the year in each prefecture-level municipality; capital input was expressed in terms of the actual capital stock [53,54,55,56]. As capital stock data are not easily available for each prefecture-level municipality, the perpetual inventory method was used, with each municipality’s fixed-asset investment in 2009 divided by 10% as the base period capital stock while the depreciation rate of fixed assets was uniformly set at 10.6%. Energy inputs were expressed using social electricity consumption. Output indicators were divided into desired and non-desired outputs. Expected output indicators were mainly expressed in terms of the city’s GDP at constant 2009 prices. Non-desired output indicators were expressed as a composite pollution index that combined three indicators: industrial wastewater emissions, industrial sulfur dioxide emissions, and industrial smoke (dust) emissions, calculated using the entropy method that was based on the study by Xin (2019). The global Malmquist–Luenberger (GML) production index was also used to measure the GFTP [57,58,59].
  2. Green economy. In this study, total green gross domestic product (GGDP) was used to measure the level of green economic development in cities. Referring to the measurement method of Rehfeld et al. (2007), green GDP (= 0.5 × environmental composite index + 0.5 × deflated gross industrial output) was used as the output indicator. The environmental composite index is obtained by averaging six indicators, namely industrial waste gas, industrial wastewater, industrial solid waste emissions, industrial sulfur dioxide, total industrial soot emissions, and energy consumption per unit of industrial GDP after negative normalization [60].
  3. Industrial structure (s). This study used the proportion of secondary output to total GDP to measure the industrial structure of cities. As China is currently in a period of economic growth, industrial energy consumption is significantly higher than that of other sectors, and the emissions produced by them are undoubtedly the main source of PM_2.5_. At the same time, the industrial sector is also the main application industry for green technological innovation. Therefore, this paper considered the change in the industrial structure of the city as a mediating variable to explore the role of the industrial structure in the relationship between green technological innovation and haze. The role of the industrial structure in mediating the relationship between green technological innovation and hazardous air pollutants was consequently explored.
  4. Energy conservation (es). This study used the total annual liquefied petroleum gas (LPG) supply metric to measure urban energy savings. The burning of fossil fuels is considered an important source of hazardous air pollutants, and the use of LPG contributes to the burning of fossil fuels [61], thus contributing to haze control. At the same time, one of the main applications of green technological innovation is the adoption of more advanced technologies and clean energy to reduce the use of fossil fuels. Therefore, this study considered urban energy conservation changes as a mediating variable and explored the mediating role of energy conservation between green technological innovation and hazardous air pollutants.

(4) Control variables
  1. Fiscal expenditure (pe). In this study, we used fiscal expenditure within the general budget of local governments to measure the level of fiscal expenditure in cities. Fiscal expenditure represents the government’s public expenditure, including the expenditure on haze control; therefore, the larger the public expenditure, the better the effect on hazardous air pollutants. This paper used the impact of fiscal expenditure controls on hazardous air pollutants.
  2. Transport (tra). Exhaust emissions from motor vehicles in public transport are an important source of PM_2.5_. In Beijing, Shanghai, and Tianjin, PM_2.5_ emissions from motor vehicle exhausts accounted for approximately 22%, 25%, and 16% of their total emissions, respectively. Therefore, in this study, the total annual passenger traffic of public transport was selected to control the impact of transport on hazardous air pollutants.
  3. Degree of openness to the outside world (FDI). The phenomenon of “bottom-up competition” by local governments to promote economic growth and attract large amounts of foreign direct investment (FDI) will lead to a deterioration in environmental quality. Therefore, this study used FDI to control the impact of urban development levels on hazardous air pollutants in China.

#### 2.2.2. Data Sources

After matching, the final sample comprises a set of balanced panel data from 283 cities in China for the period of 2009–2018. The sources of data and definitions of the variables are presented in Table 1. 

#### 2.2.3. Spatial Weighting Matrix

Under the influence of natural factors such as atmospheric circulation and atmospheric chemistry as well as economic mechanisms such as industrial transfer and transportation, hazardous air pollutants have a strong spatial correlation effect, making it essential to incorporate a weighting matrix reflecting spatial relationships into the model when studying hazardous air pollutants. Therefore, in this study, a geospatial weight matrix based on the number of nearest road miles between cities was constructed to reflect the influence of geographical factors on hazardous air pollutants. At the same time, a spatial weight matrix of economic distance based on urban GDP per capita was also constructed. This was performed as per Zhao (2017) for reflecting the influence of economic factors on hazardous air pollutants, which has been used for robustness testing [62].

## 3. Results

### 3.1. Spatial Econometric Model Regression Results and Analysis

#### 3.1.1. Spatial Correlation Test of Hazardous Air Pollutants

The Moran index reflects the degree of similarity between the attribute values of spatially contiguous or spatially adjacent regional units and is calculated as follows:

(4) $$I=\frac{n\sum_{i=1}^{n}\sum_{j=1}^{n}\omega_{ij}(x_{i}-\bar{x})(x_{j}-\bar{x})}{\sum_{i=1}^{n}\sum_{j=1}^{n}\omega_{ij}\sum_{i=1}^{n}{\left(x_{i}-\bar{x}\right)}^{2}}=\frac{\sum_{i=1}^{n}\sum_{j=1}^{n}\omega_{ij}(x_{i}-\bar{x})(x_{j}-\bar{x})}{S^{2}\sum_{i=1}^{n}\sum_{j=1}^{n}\omega_{ij}}$$

where n is the number of samples, x_i_ and x_j_ represent the PM_2.5_, the emissions of city i and city j, respectively, S^2^ is the variance of x_i_ or x_j_ and $\;\bar{x}\;$ is the mean of x_i_ or x_j_. Moreover, ij is an element of the spatial weight matrix indicating the proximity of cities i and j. The Moran index takes values in the range [−1, 1], with a positive number indicating the existence of a positive spatial correlation, a negative number indicating the existence of a negative spatial correlation, and 0 indicating the random distribution characteristics of space, i.e., no spatial correlation. Using 283 prefecture-level cities as the study objects and regional PM_2.5_ emissions as the observed values, the Moran indices were calculated for the years 2009–2018 (see Table 2). The Moran indices for different years passed the significance test, indicating that hazardous air pollutants at the prefecture level have significant spatial autocorrelation. Specifically, the Moran indices of PM_2.5_ emissions fluctuated in a small range above and below 0.2 during the observation period, and the spatial agglomeration was generally in a relatively stable state, indicating that the state of cities with relatively similar levels of PM_2.5_ emissions did not change any more significantly in spatial distribution during the observation period.

#### 3.1.2. Spatial Panel GS2SLS Model Regression Results and Discussion

Table 3 presents the estimation results of the spatial panel benchmark regression model GS2SLS, and columns (1) and (2) show the results of the fixed effects and random effects models, considering only the core explanatory variables and the mediating variables included in the model. Columns (3) and (4) present the estimation results after the inclusion of the control variables. The Hausman tests conducted for the regression results in each column in Table 3 all passed the test at the 1% significance level; therefore, the fixed effects model should be selected.

The coefficients of the spatial lags of hazardous air pollutants in Table 2 were all significantly positive at the 1% significance level, indicating that hazardous air pollutants had a significant spatial spillover effect, i.e., hazardous air pollutants in the region will also have affected hazardous air pollutants in the surrounding regions. Due to the atmospheric flow caused by weather factors such as wind direction, temperature difference, and rainfall, hazardous air pollutants in the region are closely related to the hazardous air pollutants in geographically close areas due to natural geographical factors. At the same time, the spatial correlation of hazardous air pollutants between regions is further strengthened by economic factors such as inter-regional industrial transfer, cross-regional commodity trade, and the externalities of environmental policies. Therefore, hazardous air pollutants in the region also impact hazardous air pollutants in the surrounding areas. As can be seen from Table 2, the regression results of the spatial econometric models in columns (1)–(4) showed a significant negative correlation between the core explanatory variable, the level of green technological innovation, and hazardous air pollutants both in terms of random effects and fixed effects. Hazardous air pollutants will show a decreasing trend as the level of green technological innovation increases.

As for the mediating variables, there was a negative relationship between the effect of green technological progress (GTFP) and hazardous air pollutants. However, its coefficient did not pass the significance test. The possible reason for this may be that green technological progress requires a certain amount of time to accumulate, and the level of green innovation in Chinese cities can only be raised to a certain level before the overall level of green progress of enterprises can be raised. It indicates that, at present, the green innovation capacity of Chinese cities needs to be further improved. There was a significant negative correlation between the energy-saving effect (es) and hazardous air pollutants, indicating that under China’s energy structure, which is still dominated by the burning of fossil fuels, the development of energy-saving technologies and the use of new energy sources can effectively reduce the burning of fossil fuels, which in turn has a mitigating effect on hazardous air pollutants. There was a significant negative correlation between the GGDP and hazardous air pollutants, suggesting that under China’s policy objective of vigorously developing a green economy, the country has further pushed Chinese enterprises with high pollution and high emissions to go green, prompting them to engage in cleaner production, thus mitigating hazardous air pollutants. However, there was a significant positive correlation between the industrial structure effect (s) and hazardous air pollutants, i.e., the industrial structure of Chinese cities is still at the stage of exacerbating hazardous air pollutants, indicating that China’s industrial structure needs to be further upgraded to a cleaner one.

As for the control variables, there was a non-significant positive correlation between foreign investment (FDI) and hazardous air pollutants, indicating that foreign investment in Chinese cities is still dominated by polluting enterprises; however, with the development of Chinese cities’ economies and the strict enforcement of government environmental policies, the proportion of “clean” foreign investment introduced gradually increases and shows a positive but non-significant contribution to hazardous air pollutants. There was a significant positive correlation between fiscal expenditure (pe) and hazardous air pollutants, probably because promotions for local officials in China use the GDP growth rate as the main performance assessment indicator. There was a significant positive correlation between the public transport situation (tra) and hazardous air pollutants, indicating that the development of public transport can effectively improve local air pollution. This is because rail transport and buses in most Chinese cities are dominated by electricity and new energy sources. The development of public transport helps to reduce private bus trips, reducing vehicle emissions from petrol and kerosene-based fossil energy combustion, which in turn would help to reduce hazardous air pollutants.

#### 3.1.3. Robustness Tests

In this study, we mainly used the methods of replacing the explanatory variables, the spatial weight matrix, and the instrumental variables to test the robustness of the baseline regression results. This was achieved by (1) replacing PM_2.5_ with PM10 to represent the air pollution variable in the regression, with PM10 data obtained from the website of the Ministry of Ecology and Environment of China; (2) replacing the geographic distance spatial weight matrix used in the previous regression with the economic distance matrix; (3) replacing the highest second-order spatial lag term used in the previous regression with the highest third-order spatial lag term based on the GS2SLS regression as the instrumental variable. The regression results show that the spatial lag term of hazardous air pollutants is still significant, and the negative relationship between the core explanatory variable of urban green technological innovation level and hazardous air pollutants was still significant. It can be observed that the previous benchmark regressions had strong robustness. The results of the robustness tests in this study are presented in Table 4.

#### 3.1.4. Sub-Regional Testing

According to the gradient development theory of regional economics, China’s economic zones are divided vertically into three major regions: east, central, and west. Considering the large differences in economic development between these three regions, to further investigate whether there is heterogeneity in the spatial spillover effect of green technological innovation on hazardous air pollutants in regions with different levels of development, this paper divided the 283 cities in the sample into three parts according to the economic zones they belong to, i.e., east, central, and west, and conducted spatial regressions respectively. The regression results are shown in Table 5.

In terms of the coefficient of the spatial lag term of hazardous air pollutants, there was a significant spatial spillover effect of hazardous air pollutants in all three regions of China (east, central, and west), all of which were significantly positive at the 1% significance level; however, the coefficient of the spatial lag term of hazardous air pollutants was the largest in the eastern region, the second largest in the central region, and the smallest in the western region. The possible reason for this is that hazardous air pollutant levels are higher in eastern China than in central and western China as trade and economic ties between eastern regions are closer than those between central and western regions. Therefore, the spatial spillover effect of hazardous air pollutants is greater in the eastern regions of China than in the central and western regions.

In terms of the core explanatory variable of green technological innovation, there was a significant negative correlation between green technological innovation and hazardous air pollutants in all three regions of China, i.e., green technological innovation helps to mitigate hazardous air pollutants. In terms of regression coefficients, green technological innovation had the greatest inhibitory effect on hazardous air pollutants in the central region, followed by the eastern region, and then the western region. The main reason for this is that the economic development of the eastern and central regions of China is higher, and the corresponding industrial structure is better, therefore the overall level of green innovation of enterprises is higher. The western region lags in terms of economic development, the level of green technological innovation is relatively low, and the level of hazardous air pollutants is also relatively low. Therefore, the inhibiting effect of green scientific and technological innovation on hazardous air pollutants in western China is not significant.

In terms of mediating variables, there was a non-significant negative correlation between the green technology effect and hazardous air pollutants in all three major regions of China, indicating that all regions of China need to further enhance the level of green technological innovation to further improve China’s green technology progress. There was only a significant negative correlation between the energy-saving effect and hazardous air pollutants in the eastern region of China, while a non-significant positive correlation existed in the central and western regions because new and clean energy technologies are more widely used in the eastern region than in the central and western regions. The industrial structure significantly contributes to hazardous air pollutants in all regions of China, which is consistent with the regression results for China as a whole, indicating that China’s industrial structure still needs further optimization. The green economy effect has a significant inhibitory effect on hazardous air pollutants in all regions of China, which is consistent with the regression results for China as a whole, indicating that China should continue to vigorously develop the green economy and use the development of the green economy to drive up the level of urban haze control.

In terms of control variables, there was a negative relationship between openness to the outside world and hazardous air pollutants in the eastern and western regions of China and a positive but insignificant relationship between openness to the outside world and hazardous air pollutants in the central region. There was a significant negative effect of public transport development on cities in eastern China and a non-significant positive effect on central and western China, probably because the level of urban transport infrastructure development in central and western China is lower than that in eastern China. Fiscal spending had a significant negative correlation with hazardous air pollutants in eastern China, while a non-significant positive correlation existed in the central and western regions. This suggests that the “bottom-up” effect of the pressure of “GDP” growth and hazardous air pollutants’ control in the central and western regions still exists but has less impact on hazardous air pollutants.

#### 3.1.5. Innovative Pilot City Test

To improve the level of innovation in Chinese cities and in China as a whole, in 2008, China implemented a pilot project to build innovative cities and expanded the scope of the pilot project in an orderly manner. By the end of the study period, there were 78 pilot cities in China, but four cities, including Lhasa and Shihezi, were excluded from the study due to lack of statistical data; therefore, the total number of innovative pilot cities involved in the sample was 74. This study further divided the sample cities in the panel data into innovative and non-innovative cities and further examined the heterogeneous role of green scientific and technological innovation on hazardous air pollutants in innovative and non-innovative cities based on a spatial econometric model. The regression results for the innovative pilot cities are presented in Table 6. It can be found that, in terms of the spatial lag effect of hazardous air pollutants, the spatial spillover effect of hazardous air pollutants in innovative cities was greater than that in non-innovative cities, and green technological innovation had a significant inhibitory effect on hazardous air pollutants in both innovative and non-innovative cities. This suggests that China should further increase the construction of pilot innovative cities to accelerate the development of green innovation technology with a higher concentration of innovative talents and capital elements, and thus suppress hazardous air pollutants.

### 3.2. A Model Test of the Mediating Effect of Green Technological Innovation on Hazardous Air Pollutants

From the previous section, it can be seen that green technological innovation may affect hazardous air pollutants in four ways: green technology progress, energy-saving, industrial structure, and green economy effects. A mediating effect model consisting of the following three regression equations was constructed to identify the above transmission pathways, and the regression results are shown in Table 7.

(5) $$\ln PM_{it}=\theta_{0}+\theta_{1}\ln E_{it}+\theta_{2}Y_{it}+\zeta_{it}$$

(6) $$D_{it}=\beta_{0}+\beta_{1}\ln E_{it}+\beta_{2}Y_{it}+\mu_{it}$$

(7) $$\ln PM_{it}=\gamma_{0}+\gamma_{1}\ln E_{it}++\gamma_{2}Y_{it}+\gamma_{3}D_{it}+\tau_{it}$$

Y is the vector set consisting of control variables; D is the possible mediating variables, including industrial structure effect (lnsec), energy-saving effect (lnes), green technological progress effect (lnGTFP), and green economic effect (ln GGDP); E and PM are the levels of green technological innovation and PM_2.5_, respectively. According to the principle of the mediating effect model, if the coefficients θ_1_, θ_2_, β_1_ or β_2_, and γ_4_ are significant, and the coefficients γ_1_ and γ_2_ become smaller or less significant than θ_1_ and θ_2_, this indicates the existence of a mediating effect. The regression results of the mediating effect test for green technological innovation on hazardous air pollutants in Chinese cities are shown in Table 7.

When green technological progress is considered as a mediating variable, the coefficient of green technological innovation in Equation (5) decreases compared to Equation (7), the effect of green technological innovation on green technological progress in Equation (6) is not significant, and green technological progress in Equation (7) does not significantly contribute to hazardous air pollutants, indicating that green technological progress is not a mediating variable between green technological innovation and hazardous air pollutants in Chinese cities. A possible reason for this is that the results of green technological innovation can only be produced and applied after reasonable transfer and transformation by enterprises, and there is a certain lag between the generation of innovation patents and their actual application. Therefore, at this stage, the inhibiting effect of green technological progress on hazardous air pollutants was not significant. When energy conservation is regarded as a mediating variable, the coefficient of green technological innovation in Equation (5) decreases compared to that in Equation (7), and green technological innovation has a significant promoting effect on energy conservation in Equation (6). Energy conservation has a significant inhibiting effect on hazardous air pollutants in Equation (7), indicating that energy conservation is a mediating variable between green technological innovation and hazardous air pollutants, i.e., green technological innovation can reduce hazardous air pollutants through the energy conservation effect. When the industrial structure is considered as a mediating variable, the coefficient of green technological innovation in Equation (5) decreases compared to Equation (7), but the effect of green technological innovation on industrial structure in Equation (6) is not significant, indicating that the industrial structure of Chinese cities is not a mediating variable between green technological innovation and hazardous air pollutants. A possible reason for this is that the traditional industrial sector is still contributing more to China’s economic growth, and the upgrading of the industrial structure still needs a long-term process to have a significant inhibiting effect on hazardous air pollutants. When the green economy is considered as a mediating variable, the coefficient of green technological innovation in Equation (5) decreases compared with that in Equation (7), while the green technological progress in Equation (6) has a significant promoting effect on the green economy, and the green economy in Equation (7) plays a significant inhibiting role on hazardous air pollutants, indicating that the green economy is a mediating variable between green technological innovation and hazardous air pollutants; that is, green technological innovation can reduce hazardous air pollutants through the green economic effect to reduce hazardous air pollutants.

Therefore, the following conclusions can be drawn. The energy-saving effect and the green economic effect have a mediating effect on the role of innovation efficiency in hazardous air pollutants. It can be seen that in the process of improving the level of green innovation in cities, the haze reduction effect of green technological progress and industrial structure optimization has failed to act effectively, which is an important reason for the increase in hazardous air pollutants in China.

## 4. Discussion

The results are consistent with the existing literature [25,30], which found a negative relationship between innovation and pollution in China. In fact, this is because green scientific and technological innovation takes green development as its core pursuit, focusing on providing new products, processes, services, and market solutions through innovation, thus reducing natural resource consumption, reducing ecological and environmental damage, improving resource allocation efficiency, and helping the economy to move towards higher quality development. At present and on a practical level, through a combination of independent research, development, and the introduction of technology, China’s green technology level in the ecological and environmental fields has been continuously improving, and the green technology gap with the international advanced level has continued to narrow. The proportion of key technologies and equipment domestically produced in China is increasing, and the world’s largest industrial supply capacity has been formed in the fields of dust removal, flue gas desulfurization, and air pollution treatment. The products of leading technologies can meet the needs of current environmental pollution treatment. Therefore, the empirical regression results of this study also prove once again that green technological innovation in Chinese cities can help reduce hazardous air pollutants.

This paper shows that the western region, where low-end manufacturing occupies a high proportion of the industrial structure, does not pay enough attention to environmental governance, resulting in a relatively high hazardous air pollutants level. Hence, the government should strengthen the supervision of enterprises’ emissions and environmental protection assessments to force them to change their energy structure, increase the use of new energy sources, and strengthen the upgrading of their emissions technology. Pollution emission standards must be improved, stricter pollutant emission limits for key industries in key regions should be implemented, and localities should be encouraged to develop and implement local pollutant emission standards that are stricter than national standards. The enterprise, as the main body of the industrial technological innovation mechanism, should be improved, making enterprise demand-oriented to promote the realization of a rapid and effective transformation of scientific and technological achievements [63]. The development of large energy-saving and environmental protection enterprises should be cultivated to improve the industry’s research and development capabilities, level of specialization, and technical level. Compared to the western region, the spatial spillover effect of hazardous air pollutants in the central and eastern regions is greater because of factors such as the dense population in the central and eastern regions, contiguous regional economic growth, significant industrial agglomeration, and the transfer of polluting industries. In comparison, the western region is less densely populated, has less economic synergy, and undertakes less transfer of polluting industries from the central and eastern regions, therefore the spatial spillover effect of hazardous air pollutants is lower.

## 5. Conclusions

Using panel data of 283 prefecture-level cities in China from 2009 to 2018, this paper systematically examined the relationship between green technological innovation and hazardous air pollutants by employing a spatial econometric model based on GS2SLS. Further, this paper explored the differences in the impact of green scientific and technological innovation on hazardous air pollutants at different stages of economic development and its transmission mechanism. The results show that hazardous air pollutants have significant spatial spillover effects, i.e., hazardous air pollutants in the region also affect hazardous air pollutants in surrounding areas. The spatial spillover effects of hazardous air pollutants are greater among innovative cities than in non-innovative cities, and thus their green innovation levels should be further improved. In terms of the direct impact of green scientific and technological innovation on hazardous air pollutants, the level of green scientific and technological innovation in China as a whole and in the eastern and central regions has a significant inhibitory effect on hazardous air pollutants.

Although this research analyzed the relationship between green technological innovation and hazardous air pollutants, the following are notable. First, the selection of indicators in this paper is not yet very comprehensive, and only a simple selection has been made without in-depth research. It is hoped that in future research, this aspect of research can be continuously improved and deepened to provide a reference basis and application value for the government. Second, the empirical analysis in this paper only attempts the GS2LS model. A non-linear relational model could be used in further research.

## Figures and Tables

**Table 1:** Variable descriptions and data sources.

Variable Type	Indicator Selection	Indicators	Data Sources
Explained variables	Hazardous air pollutants	Annual average PM_2.5_ concentration	International Earth Science Information Network, Columbia University https://beta.sedac.ciesin.columbia.edu/ (accessed on 10 September 2020)
Core explanatory variables	Green technology level	Total annual green patent applications	National Intellectual Property Office https://www.cnipa.gov.cn/ (accessed on 10 September 2020)
Intermediate variables	Energy savings	Total annual LPG gas supply	“City Statistics Yearbook 2009–2018”
	Green economy	Green GDP	Using a composite environmental index weighted by gross industrial product
	Technological advances	Green total factor productivity	Measured by GML method
	Industrial structure	Secondary output as a share of GDP	“City Statistics Yearbook 2009–2018”
Control variables	Financial expenditure	Local finance general budget expenditure	“City Statistics Yearbook 2009–2018”
	Transportation	Total bus passenger traffic for the year	
	Degree of openness to the outside world	Actual use of foreign direct investment	

**Table 2:** Moran index of PM_2.5_ emissions for 283 cities in China, 2009–2018.

**Year**	**2009**	**2010**	**2011**	**2012**	**2013**
Moran’I	0.199	0.202	0.196	0.205	0.201
*p*-value	0.000	0.000	0.000	0.000	0.000
**Year**	**2014**	**2015**	**2016**	**2017**	**2018**
Moran’I	0.196	0.196	0.201	0.199	0.210
*p*-value	0.000	0.000	0.000	0.000	0.000

**Table 3:** Spatial GS2SLS model regression results.

Variables	The Geographical Distance-Based Spatial Weighting Matrix			
	(1)	(2)	(3)	(4)
	FE	RE	FE	RE
W1∗lnpm25	2.213 ***	1.413 ***	2.239 ***	1.431 ***
	(0.055)	(0.037)	(0.054)	(0.036)
lnE	−0.015 ***	−0.050 ***	−0.013 ***	−0.044 ***
	(0.004)	(0.003)	(0.004)	(0.004)
lnGTFP	−0.094	−0.116	−0.094	−0.106
	(0.069)	(0.076)	(0.069)	(0.078)
lnes	−0.002 *	−0.003 **	−0.002 **	−0.003 **
	(0.001)	(0.002)	(0.001)	(0.002)
lnsec	0.012 *	0.026 ***	0.019 ***	0.025 ***
	(0.006)	(0.007)	(0.006)	(0.007)
lnGGDP	−0.014 **	−0.030 ***	−0.014 **	−0.027 ***
	(0.006)	(0.006)	(0.006)	(0.007)
lnFDI			0.002	0.004
			(0.002)	(0.003)
lntra			−0.016 **	−0.025 ***
			(0.007)	(0.008)
lnpe			0.007 **	−0.003
			(0.003)	(0.005)
Adjusting R^2^	0.638	0.638	0.698	0.698
Wald test (p)	5395.518	4104.138	6341.145	4333.550
	(0.000)	(0.000)	(0.000)	(0.000)
Hausman test (p)	298.020 (0.000)		268.534 (0.000)	

Note: ***, **, * denote significance levels of 1%, 5%, and 10%, respectively; values in square brackets below the coefficients are standard errors; FE and RE denote fixed-effect models and random effect models, respectively.

**Table 4:** Robustness Tests.

	(1)	(3)	(4)
W1∗lnpm25	2.240 ***	2.234 ***	2.235 ***
	(0.055)	(0.054)	(0.056)
lnE	−0.011 ***	−0.012 ***	−0.011 ***
	(0.004)	(0.004)	(0.004)
lnGTFP	−0.094	−0.095	−0.098
	(0.070)	(0.069)	(0.070)
lnes	−0.002 **	−0.002 **	−0.002 **
	(0.001)	(0.001)	(0.002)
lnsec	0.018 ***	0.018 ***	0.019 ***
	(0.007)	(0.006)	(0.007)
lnGGDP	−0.014 **	−0.014 **	−0.014 ***
	(0.006)	(0.006)	(0.007)
lnFDI	0.002	0.002	0.004
	(0.002)	(0.002)	(0.003)
lntra	−0.017 ***	−0.017 **	−0.018 ***
	(0.008)	(0.007)	(0.008)
lnpe	0.007 **	0.007 **	0.007 ***
	(0.003)	(0.003)	(0.002)
Adjusted R^2^	0.632	0.698	0.704
Wald test (p)	5393.466	6340.145	4356.834
	(0.000)	(0.000)	(0.000)

Note: *** and ** denote significance levels of 1% and 5%, respectively; values in brackets below the coefficients are standard errors.

**Table 5:** Regression results by region.

Explanatory Variables	Geographical Distance Spatial Weighting Matrix		
	Eastern Region	Central Region	Western Region
W1∗lnpm25	6.201 ***	5.253 ***	2.632 ***
	(0.258)	(0.349)	(0.378)
lnE	−0.035 ***	−0.009 ***	−0.002
	(0.006)	(0.003)	(0.016)
lnGTFP	−0.052	−0.101	−0.110
	(0.084)	(0.139)	(0.130)
lnes	−0.004 **	0.008 ***	0.002
	(0.002)	(0.003)	(0.002)
lnsec	0.017 ***	0.113 ***	0.027 **
	(0.006)	(0.035)	(0.014)
lnGGDP	−0.021 ***	−0.030 ***	−0.011 ***
	(0.009)	(0.012)	(0.001)
lnfdi	−0.002	0.004	−0.001
	(0.003)	(0.006)	(0.004)
lntra	−0.012	−0.004	−0.035 ***
	(0.009)	(0.017)	(0.012)
lnpe	−0.042 ***	0.028	0.004
	(0.013)	(0.021)	(0.004)

Note: *** and ** denote significance levels of 1% and 5%, respectively; values in brackets below the coefficients are standard errors.

**Table 6:** Regression results for innovative pilot cities.

Explanatory Variable	Geographical Distance Spatial Weighting Matrix (*W_1_*)	
	Innovative Cities	Non-Innovative Cities
W1∗lnpm25	4.352 ***	2.391 ***
	(0.289)	(0.085)
lnE	−0.052 ***	−0.005 *
	(0.011)	(0.003)
lnGTFP	0.178	0.148
	(−0.136)	(−0.094)
lnes	0.001	0.002
	(0.003)	(0.002)
lnsec	0.026	0.010
	(0.018)	(0.008)
lnGGDP	−0.016	−0.027 ***
	(0.016)	(0.008)
lnfdi	−0.008	−0.006 *
	(0.006)	(0.006)
lntra	−0.021	−0.034 ***
	(0.021)	(0.009)
lnpe	0.005	0.007
	(0.006)	(0.008)

Note: *** and * denote significance levels of 1% and 10%, respectively; values in brackets below the coefficients are standard errors.

**Table 7:** Testing the mediating effect of green technological innovation on hazardous air pollutants.

**Variables**	**D = lnGTFP**			**D = lnes**		
	**Equation (5)**	**Equation (6)**	**Equation (7)**	**Equation (5)**	**Equation (6)**	**Equation (7)**
lnE	−0.010 *** (0.003)		−0.013 *** (0.004)	−0.010 *** (0.003)		−0.013 *** (0.004)
D		0.003 * (0.002)	−0.094 (0.069)		−0.093 ** (0.040)	−0.002 ** (0.001)
**Variables**	**D = lnsec**			**D = lnGGDP**		
	**Equation (5)**	**Equation (6)**	**Equation (7)**	**Equation (5)**	**Equation (6)**	**Equation (7)**
lnE	−0.010 *** (0.003)		−0.013 *** (0.004)	−0.010 *** (0.003)		−0.013 *** (0.004)
D		−0.006 (0.007)	−0.014 ** (0.006)		0.135 *** (0.009)	−0.019 *** (0.006)

Note: ***, ** and * denote significance levels of 1%, 5% and 10%, respectively; values in brackets below the coefficients are standard errors.

## Data Availability

No new data were created or analyzed in this study. Data sharing is not applicable to this article.
